# Wearable Inertial Gait Algorithms: Impact of Wear Location and Environment in Healthy and Parkinson’s Populations

**DOI:** 10.3390/s21196476

**Published:** 2021-09-28

**Authors:** Yunus Celik, Sam Stuart, Wai Lok Woo, Alan Godfrey

**Affiliations:** 1 Department of Computer and Information Sciences, Northumbria University, Newcastle upon Tyne NE1 8ST, UK; yunus.celik@northumbria.ac.uk; 2 Department of Sport, Exercise and Rehabilitation, Northumbria University, Newcastle upon Tyne NE1 8ST, UK; sam.stuart@northumbria.ac.uk

**Keywords:** gait analysis, wearable electronic devices, computing methodologies, patient outcome assessment

## Abstract

Wearable inertial measurement units (IMUs) are used in gait analysis due to their discrete wearable attachment and long data recording possibilities within indoor and outdoor environments. Previously, lower back and shin/shank-based IMU algorithms detecting initial and final contact events (ICs-FCs) were developed and validated on a limited number of healthy young adults (YA), reporting that both IMU wear locations are suitable to use during indoor and outdoor gait analysis. However, the impact of age (e.g., older adults, OA), pathology (e.g., Parkinson′s Disease, PD) and/or environment (e.g., indoor vs. outdoor) on algorithm accuracy have not been fully investigated. Here, we examined IMU gait data from 128 participants (72-YA, 20-OA, and 36-PD) to thoroughly investigate the suitability of ICs-FCs detection algorithms (1 × lower back and 1 × shin/shank-based) for quantifying temporal gait characteristics depending on IMU wear location and walking environment. The level of agreement between algorithms was investigated for different cohorts and walking environments. Although mean temporal characteristics from both algorithms were significantly correlated for all groups and environments, subtle but characteristically nuanced differences were observed between cohorts and environments. The lowest absolute agreement level was observed in PD (ICC_2,1_ = 0.979, 0.806, 0.730, 0.980) whereas highest in YA (ICC_2,1_ = 0.987, 0.936, 0.909, 0.989) for mean stride, stance, swing, and step times, respectively. Absolute agreement during treadmill walking (ICC_2,1_ = 0.975, 0.914, 0.684, 0.945), indoor walking (ICC_2,1_ = 0.987, 0.936, 0.909, 0.989) and outdoor walking (ICC_2,1_ = 0.998, 0.940, 0.856, 0.998) was found for mean stride, stance, swing, and step times, respectively. Findings of this study suggest that agreements between algorithms are sensitive to the target cohort and environment. Therefore, researchers/clinicians should be cautious while interpreting temporal parameters that are extracted from inertial sensors-based algorithms especially for those with a neurological condition.

## 1. Introduction

Human gait is a complex cyclic pattern that relies on individuals′ kinetic, kinematic and muscle characteristics. Neurodegenerative disorders (e.g., Parkinson’s disease, PD) and other factors like age and lifestyle can altering an individual’s gait pattern [1]. Typically, people with PD walk slowly with short fast shuffling steps [2,3]. Additionally, those with PD may present with additional conditions due to poor gait such as pain arising poor foot health and reduced quality of life [4] leading to increased depression scores [5]. Although most neurological conditions share similar gait deficits such as reduced gait speed and poor balance, there are also characteristically distinctive patterns (e.g., increased step time) that help differentiate particular neurological conditions [6]. Therefore, investigating discrete gait cycles may provide nuanced and even personalized assessments for those with gait disturbances.

Wearable inertial measurement units (IMUs) are now commonly used for gait analysis due to their small form factor and long data recording possibilities, in indoor and outdoor environments [7,8]. The vertical acceleration of the pelvis and sagittal plane angular velocity of the shins are commonly used inertial signals to detect initial contact (IC) and final contact (FC) within the gait cycle [9,10,11]. In general, methods to quantify ICs and FCs are dependent upon inertial signal quality as well as IMU location (e.g., lower-back, shin/shank, foot) and computational methodology (e.g., wavelet transform) [6,9,10,11].

Research demonstrates that either linear acceleration or angular velocity sensors attached to various body locations/segments can be used to detect ICs-FCs as accurately as a reference system (e.g., footswitches, instrumented walkway) for both normal and pathological gait footfalls [12,13,14,15,16,17,18,19,20,21]. However, accuracy of IMU algorithm also varies depending on walking terrain (environment) and target population. Previous studies investigated performance of IMU algorithms that provide accurate and repeatability valid ICs-FCs. For example, lower-back algorithms that use acceleration signals were compared in healthy [22,23] and neurological populations during indoor walking [24]. Wrist, waist and shank accelerometer signal-based algorithms were compared during various walking settings (e.g., indoor, outdoor) in a healthy young population [25]. Performances of foot and shank angular velocity with foot acceleration signal-based algorithms were compared in spinal-cord injured individuals [19]. Other studies investigated optimal IMU locations (lower-back, shank, foot) and algorithms that provide accurate ICs-FCs moments for healthy young adults only [9,11]. Each study reported various levels of accuracy, where inconsistencies could be associated with the fluctuations in performances of IMU algorithms e.g., better detecting ICs than FCs [25] due to the higher variance of generated signals by each cohort during walking on different terrains [11].

Performances of lower-back IMU algorithms are typically poorer/lower in neurological cohorts compared to healthy cohorts, due to occasional failed detection of acceleration-based ICs-FCs [24]. This could be attributed to the development of the algorithms within controlled environments only [9]. Moreover, previous studies reported certain differences between indoor and outdoor temporal parameters [2,26,27,28] and this was associated with the fluctuation in performances of inertial algorithms along with many other factors such as the white coat effect [29]. Indeed, previous papers investigated and compared IMU algorithms based on sensor location and target signal used by using a reference system in healthy populations [9,10,11], but the margin of error between algorithms (or absolute agreement) has not been fully investigated in different groups and environment. Furthermore, the population size of validation and comparison studies were generally limited/low. Consequently, optimal algorithms, IMU locations for a specific cohort and environment to inform how cautious researchers should be while interpreting temporal parameters remain unclear.

The aim of this study is to investigate the level of agreement between established lower-back and shank IMU algorithms in young adults (YA), older adults (OA) and PD cohorts during different walking protocols in various environments. Our hypothesis is that existing inertial algorithms may be sensitive to sensor wear location, target cohort and walking environments limiting the widespread use of wearable IMU algorithms during indoor and outdoor gait assessment. Discovering the effects of cohort and environment could help better understanding the difference between indoor and outdoor walking. Unlike previous studies, this study directly investigates agreement between algorithms rather than agreement with a reference system in large healthy and PD populations. Accordingly, we aim to make a judgement about how confidently researchers can use one algorithm over the other. The results of this study will add to the current knowledge by providing details about how similar the results of two common IMU algorithms are in various environments. To the author′s knowledge, this is the first comparative study that investigates the level of agreement between lower-back and shank sensor-based algorithms on adults and PD along with a large YA population. The main contributions are to:

(i) Investigate agreement between algorithms across different groups (YA-OA-PD),
(ii) Investigate impact of walking environment (treadmill-indoor-outdoor) on agreement between algorithms,
(iii) Provide recommendations when deciding optimal IMU location and gait algorithms.

## 2. Materials and Methods

A total of 128 participant’s gait data were analyzed from previously created datasets. Public dataset 1 (DS1 http://gaitanalysis.th-brandenburg.de/ accessed 5 October 2020) contained 72 healthy young adults (YA) [30]. Additional dataset 2 (DS2) comprises 20 (age matched) healthy older adults (OA) and 36 PD participants, a sample from a previous study [31]. See Table 1 for participant information and demographics and associated references for in-depth details Here, datasets are described briefly.

### 2.1. Datasets

#### 2.1.1. Datasets-1 (DS1)

**Figure 1:**
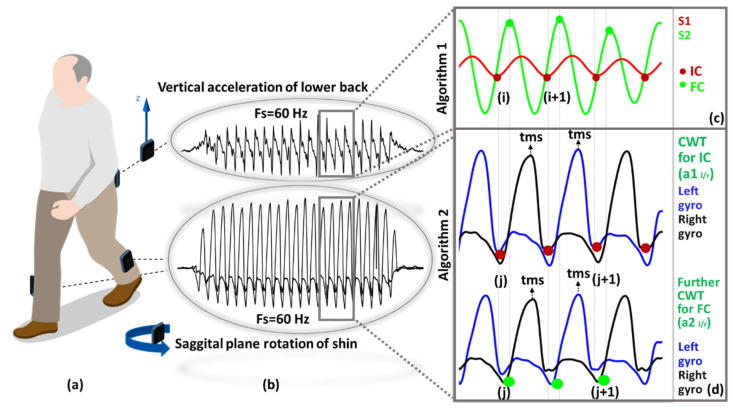
Data processing: (**a**) Sensor placement, (**b**) raw acceleration and rotation data of two different location, (**c**) IC-FC detection with Algorithm S1 (in Supplementary Material) process, (**d**) IC-FC detection with Algorithm S2 (in Supplementary Material), ICs and FCs are represented with red and green dots, respectively.

Data capture took place in different countries (Austria, Finland, Kenya) and testing environments (treadmill, indoor and outdoor). All volunteers provided informed consent about the experiments, data storage and the future use of data before participating. Comprehensive information on protocols, data collection, etc., is provided elsewhere [30]. In short, each subject wore three IMUs (Xsens MTw, Enschede, Netherlands) on right shank (SR), left shank (SL) and the lower back (fifth lumbar vertebrae, L5), Figure 1a. Each synchronized Xsens IMU was configured for different protocols (acceleration ±16 g, angular velocity ±2000 deg/s and different sampling rates: 60 Hz, 75 Hz, 100 Hz) prior to data collection.

During treadmill walking, participants were asked to walk between 7–9 min (approx. 700 m). The speed was incremented every minute from 2–8 km/h with a step of 1 km/h. During repetitive indoor walking, participants walked 10–20 m four times at self-selected normal, slow, and fast speeds. The outdoor walking experiments consisted of two 40–80 m walks at a self-selected speed.

#### 2.1.2. Datasets-2 (DS2)

Each subject wore three synchronized IMUs (Opal, V2 APDM Inc., Portland, OR, USA) located on the SR, SL and the L5 via a belt strap, Figure 1a. Each recorded tri-axial acceleration (±2 g or 6 g, 128 Hz) and tri-axial angular velocity (±1500 deg/s). Gait assessment and instrumentation were carried out by a physiotherapist and trained researchers, respectively. Ethical consent was granted by the Oregon Health & Science University institutional review board (REF: 9903). All participants gave informed written consent before participating. Repetitive indoor/lab gait tasks included: walking back and forth over 10 m for 2 min at normal/self-selected speed.

### 2.2. Methodology

Two previously validated Algorithms A1 and A2 [23,32] were used for IC-FC detection. Both use a wavelet approach to process IMU signals but have fundamental differences such as signal (acceleration vs. angular velocity) and locations (waist vs. shank). Each anatomical segment of the human body has a characteristic movement pattern and thus produces distinct acceleration and angular velocity signals. Consequently, selection of an appropriate mother wavelet is appropriate to best interpret and quantify characteristics from an IMU signal produced by the movement of a particular body segment. Custom programs (MATLAB^®^ 2019, MathWorks Inc., Natick, MA, USA) analyzed raw (sample level) IMU data for ICs-FCs detection and temporal analysis.

#### 2.2.1. Algorithm S1 (A1): Lower Back

A1 (see Supplementary Material) uses the vertical acceleration signal generated with the movement of the hip during walking. First, the tri-axial accelerometer signals were transformed to the horizontal-vertical coordinate system from sensor reference frame using an approximation algorithm [33] and low-pass filtered (4th order Butterworth, cut-off frequency 20 Hz). Then, wavelet transform: (i) numerically integrated (*cumtrapz*) and then differentiated vertical acceleration using a first order Gaussian (*gaus1*) continuous wavelet transform at scale 10 were used to detect the IC events (the local minima) (ii) further differentiated to find the FC events (local maxima), Figure 1c.

#### 2.2.2. Algorithm S2 (A2): Shanks (Right and Left)

A2 (see Supplementary Material) uses the sagittal plane rotation of shin during walking. First, wavelet decomposition 5th order Coiflets (*coif*) at 10 scales split the angular velocity signal into low and high frequency components. Then, drift and high-frequency movement artefacts were removed with an initial approximation. Afterwards, two new approximations (a1 and a2) were obtained to enhance the detection of IC/FC events. For each approximation, the time corresponding to the global maximum (tms, mid-swing) was detected. Finally, IC/FC events (negative peaks) were searched in predetermined intervals [a1: IC (tms + 0.25 s, tms + 2 s), a2: FC (tms − 2 s, tms − 0.05 s)], Figure 1d.

#### 2.2.3. Temporal Parameter and Statistical Calculations

From IC-FC moments, temporal gait characteristics were calculated. Among all temporal characteristics, only step time calculation requires both right and left foot ICs-FCs moments. Therefore, right, and left foot’s step times were calculated using time stamp information. Temporal calculation formulas are presented in Supplementary Materials (Table S1) for the left side only as the same approach is used for the right side. Temporal characteristics of both sides are then used to calculate mean, variability, and asymmetry results.

Agreements between two algorithms on the temporal parameters were evaluated using Pearson’s (r), Spearman’s (rho) and interclass correlation coefficients (ICC_2,1_) with upper and lower bounds and calculated using a two-factor mixed model to assess the level of absolute agreement (between A1 and A2) [34]. A coefficient value of ≤0.30 indicates no agreement, 0.31 to 0.50 reflects fair, 0.51 to 0.70 moderate, 0.71 to 0.90 substantial, and ≥0.91 indicates very good agreement [35,36]. Graphical analysis was performed using Bland and Altman plots [37]. Absolute differences were calculated as AD = (|A1−A2|). All statistical analyses were performed using IBM^®^ SPSS^®^ Statistics 26.

## 3. Results

Generally, algorithms provided similar results for mean temporal characteristics but with small AD. Higher agreement was found on mean compared to variability and asymmetry characteristics in all cohorts and environments.

### 3.1. A1 vs. A2: Treadmill

Agreement was substantial to very good for mean: stride time, step time and stance time, shown in Table 2. Agreement was moderate for mean swing time. Agreement for stride and step times variability was substantial to very good but fair to moderate for stance time variability and poor for swing time variability. Asymmetry parameters did not show any significant correlation except for stride time (r-rho > 0.40, ICC_2,1_ > 0.50), shown in Table 1. There were small ADs for mean stride time (0.004 s), stance time (0.001 s), swing time (0.003 s) and step time (0.004 s). Comparing overall AD and correlation coefficients between stride-step parameters and stance-swing parameters revealed that latter parameters experience larger AD and lower correlation coefficients. The AD of standard deviation in mean temporal parameters did not show any significant values.

### 3.2. A1 vs. A2: Indoor

Absolute agreements between temporal characteristics extracted using A1 and A2 during indoor walking varied for YA, OA and PD, shown in Table 3. Agreement was very good for YA, OA and PD mean stride and step times. There was substantial to very good (YA), moderate to substantial (OA and PD) agreements for mean stance and swing times.

Agreements between A1 and A2 for variability and asymmetry temporal parameters were poor. There were small ADs in mean stride, stance, swing, and step times for YA (0.017 s, 0.029 s, 0.014 s, 0.010 s), OA (0.002 s, 0.009 s, 0.003 s, 0.009 s) and PD (0.015 s, 0.022 s, 0.004 s, 0.010 s), respectively. Absolute agreement for temporal characteristics during indoor walking were highest in YA and lowest in PD. Comparing overall AD and correlation coefficients between stride-step parameters and stance-swing parameters revealed larger differences and lower correlation coefficients in the latter.

### 3.3. A1 vs. A2: Outdoor

Agreement was very good for mean stride, stance, and step times and substantial for mean swing time. Agreement between A1 and A2 for variability of stride times was moderate and fair for stance times. Remaining variability and asymmetry characteristics did not show any significant correlation. AD found 0.004 s, 0.001 s, 0.003 s, 0.004 s for mean stride, stance, swing, and step times, respectively. Differences are larger and correlation coefficients are lower in mean stance-swing times compared to mean stride-step times during outdoor walking. The data was showed in Table 4.

## 4. Discussion

To the author’s best knowledge, this is the first study to comprehensively investigate agreement levels between lower back and shank IMU algorithms. This study aimed to reveal the suitability of lower back and shank inertial algorithms on various experimental walking protocols, with different cohorts and walking environments. The alterations in the performances of lower back and shank inertial algorithms in various cohorts, especially PD, has not been previously investigated. Moreover, the impacts of treadmill, indoor and outdoor walking on the agreement of both algorithms have not been revealed. Therefore, the implications of this study will contribute to the current knowledge by providing information about the similarity of lower back and shank inertial algorithm under different conditions. The statistical results presented in this study will also shed light on future studies regarding how cautious researchers should be while interpreting results belonging to a particular environment (e.g., indoor-outdoor), cohort (e.g., PD) or temporal parameter (e.g., stance time).

Overall, location and algorithm pairs provided highly correlated mean temporal results for all cohorts during treadmill, indoor and outdoor walking. However, this is not true for variability and asymmetry characteristics. These findings attest to the common knowledge that variability and asymmetry values extracted from inertial algorithms differ across wear location [38]. This could be associated with the fact that errors or systematic delays in ICs-FCs detection affect variability measures more than mean values [39]. Our findings also suggest that agreement between location/algorithm are sensitive to age, neurological condition, and walking environment. Our results are deemed suitable for exploratory investigation as they are derived from previously validated algorithms.

### 4.1. Impact of Pathology and Age

Lowest agreement with largest AD between algorithms was in PD compared to YA and OA during indoor walking for mean, variability, and asymmetry. A previous study reported global performances of lower back IMU algorithms decreases when applied to a neurological group [24], which supports our similar findings for lower agreement. Among underlying reasons for this limitation, missing or detecting extra ICs-FCs is the most likely cause [24]. Given gait abnormalities affect the movement patterns of hip and shank segments to cause disrupted inertial waveforms [6,24], decreases in performance/agreement levels are likely. Furthermore, existing IC-FC algorithms were developed and validated for healthy populations only [9,23,32]. Disagreement was at its highest level for stance-swing time characteristics that rely on both ICs-FCs moments, aligning with previous findings [24] where A1 [23] returns greater (extra) FCs moments, thereby reducing accuracy and repeatability.

Age also affects algorithm accuracy for ICs-FCs. A study investigated age on mean, asymmetry and variability gait characteristics using chest and lower back algorithm and reported more accurate results for YA compared to OA [38]. Similarly, comparing mean temporal parameters of YA and OA during indoor walking in this study revealed agreement between algorithms are higher on YA than OA, shown in Table 3.

**Figure 2:**
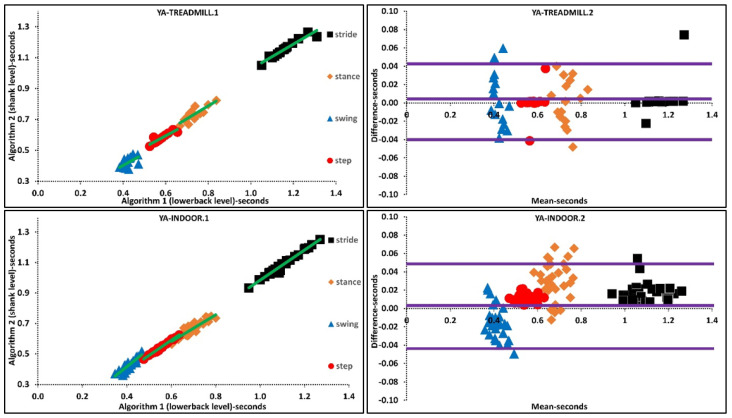
Scatter and Bland-Altman plots of algorithms 1 and 2 for investigating the agreements in older adults (OA) and PD populations by pooling all temporal parameters. OA1, PD1 are scatter plot with regression line (green), respectively. OA2, PD2 are Bland-Altman plotting with mean, lower and upper bands (purple), respectively.

**Figure 3:**
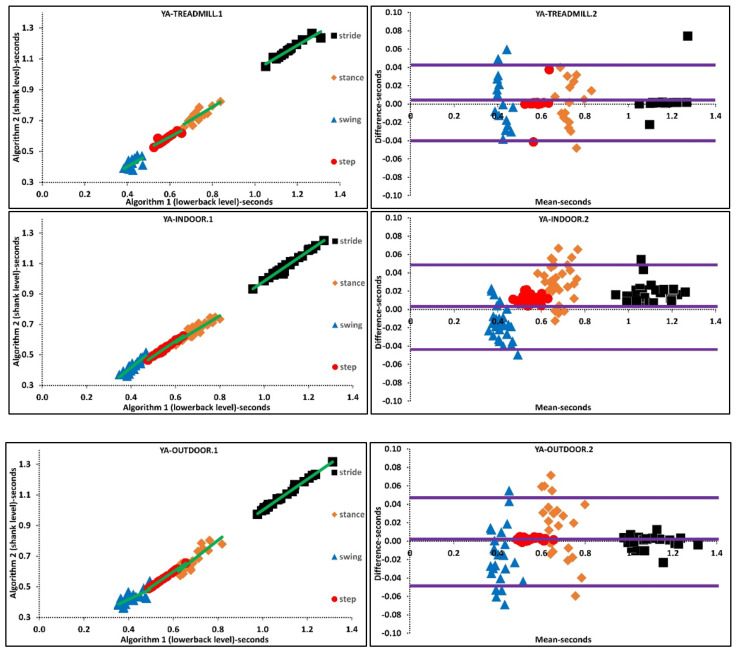
Scatter and Bland-Altman plots of algorithms 1 and 2 for investigating the agreements in various walking environments by pooling all temporal parameters. TREADMILL.1, INDOOR.1, OUTDOOR.1 are scatter plot with regression line (green), respectively. TREADMILL.2, INDOOR.2, OUTDOOR.2 are Bland-Altman plotting with mean, lower and upper bands (purple), respectively.

The above was further investigated with regression analysis, Supplementary Materials, Figure 2. For example, more ordinated regression lines were present in OA than PD. Higher agreement was observed in Bland-Altman plots where the difference axis experienced significantly lower values for OA than PD. Similarly, more ordinated regression lines were present in YA than OA. Higher agreement was observed in Bland-Altman plots where the difference axis experienced lower values for YA than OA, Figure 2 and Figure 3.

### 4.2. Impact of Environment

Various agreement levels were observed in mean, variability and asymmetry characteristics during treadmill, indoor and outdoor walking. Agreement in stride and step times is slightly higher during outdoor whereas agreement in stance and swing times is slightly higher during indoor walking. Studies have shown differences in characteristics between indoor and outdoor using IMU sensors [40,41].There are several factors that could explain the differences between extracted temporal parameters during treadmill, indoor and outdoor walking. Primarily, treadmills are classed as an external cue; forcing the person to walk to the set speed of the device, rather than having the freedom to select their own walking pattern/style. Therefore, walking on a treadmill requires additional balance skills with respect to overground walking, and harnesses or treadmill bars have an impact on patients perception and pro-prioception during walking [42]. Daily life and laboratory gait are also different, and this is associated with participants being more conscious of measurements being taken during a laboratory walking compared to free-living, which reflects more about real-life e.g., with natural dual-tasking [40]. Another factor that could explain the difference between indoor and outdoor walking is the walking terrain used (e.g., carpet, cobble) [6]. This was further studied and reported that gait adaptations strategies to maintain stability are sensitive to different walking surfaces, meaning different gait patterns are employed while walking on soft and hard terrains [43]. Given the fact that there are characteristic differences between the treadmill, indoor and outdoor walking, a previous study hypothesized that the environment plays an important role in generating different walking signals, influencing the accuracy of ICs-FCs detection [11].

Based on the findings, we suggest that the instability of IMU algorithm performances could also be a prominent reason that accounts for differences between indoor and outdoor mean characteristics. Furthermore, agreement between algorithms for variability of temporal parameters during treadmill walking is higher than indoor/outdoor walking. A higher agreement between algorithms could be associated with the fact that the treadmill as an external cue reduces variability by means of controlling walking belt speed. These results are valid for different walking speeds since treadmill walking and indoor walking experiments performed at various walking speeds. Regression and Bland-Altman plots belonging to various walking environments suggests that the difference between mean temporal parameters is lower during treadmill walking than indoor-outdoor walking, Supplementary Material, Figure 3.

### 4.3. Considerations: Sensor Location and Algorithms

Systematic delays, errors and inconsistencies in IC′s-FC′s detection are present even between two reference systems such as treadmill and motion analysis [10]. Therefore, it is crucial to investigate the level of error (agreement) between two or more IMU algorithms and minimize inconsistencies to achieve a reliable and robust methodology.

Using different IMU systems and processing methods are possible factors accounting for inconsistencies [11]. Previous studies investigated the listed factors and their impacts on the accuracy of the results on healthy subjects [9,10,11]. Here, we studied these factors in YA, OA and PD and merged with previous findings to provide a guide for future studies.

- The first factor needing consideration for IMU gait algorithms is the preferred pre-processing and post-processing methodologies as it has an impact on the extracted mean, variability, and asymmetry of temporal characteristics. For example, using algorithms like A1 [23] requires strict filtering and may affect variability of extracted characteristics as the signal is much smoother compared to less strict filters (e.g., A2).
- Sensor location and sensor signal are other important factors affecting accuracy. Research suggests the shank angular velocity signals provide more accurate and repeatable results for IC-FC detection compared to algorithms that use waist acceleration [9,10]. However, this has not been fully investigated in neurological cohorts. Here we also found that correlation/agreement of lower back and shank algorithms change when applied in various walking environments and decrease when applied to those with PD.
- Although findings show that the threshold/rule-based inertial algorithms for ICs-FCs detection provide highly correlated mean results, the fact that performances are sensitive to target cohort and environment limits widespread use.

### 4.4. Limitations and Future Works

Despite the algorithms being previously validated against reference standards (e.g., instrumented walkways), it remains a limitation that we did not collect and compare reference data in this study. However, study results are deemed suitable as validated algorithms and high-grade wearable IMUs were used, showing good agreement with previous studies [7,8,44,45,46], and the purpose here is to compare between algorithms. However, systematic errors (e.g., delays) exist in the algorithms, 0.006 s and −0.029 s were reported for ICs and FCs, respectively in the lower back algorithm whereas 0.01 s in IC detection was reported for the shank-based algorithm [23,32]. Systematic delays in ICs-FCs detection may increases in OA and PD populations due to the change of the acceleration and angular velocity of the hip and lower limb [11,24]. Given the importance of accurate ICs-FCs detection in gait analysis, more reliable and robust algorithms are needed, especially for gait assessment of neurological conditions. Moreover, wearable sensor-based gait assessment is shifting from supervised environments (e.g., lab) to unsupervised environments (e.g., free-living) because the latter enable habitual data capture [47]. Therefore, there is a need for validated inertial algorithms to be used in unsupervised environments, however, the absence of gold/reference standard systems to validate inertial algorithms in unsupervised environments bring new challenges as the field matures [29]. Severity of gait impairment has an impact on the waveform of acceleration and angular velocity signals [6]. Therefore, more advanced approaches (e.g., machine learning, deep learning) which already have shown promising results [48,49,50] should be adopted in neurological gait studies as they work independently from signal shape and thresholds. Furthermore, use of a particular target signal e.g., vertical acceleration of the hip or sagittal plane angular velocity of the shin makes the orientation of the sensor crucial. In case of inaccurate sensor placement, the algorithms provide inaccurate results. Therefore, future studies also should aim to develop algorithms that work independently from sensor orientation.

## 5. Conclusions

Investigation of the optimal IMU algorithm for detecting ICs-FCs is a trending topic and plays a crucial role in rehabilitation studies. Overall, algorithms provided significantly correlated results for mean characteristics only on YA-OA-PD during treadmill, indoor and outdoor walking. However, findings show that the level of agreement varies in different cohorts and environments. Researchers/clinicians should interpret temporal characteristics, especially stance and swing, that are extracted from inertial algorithms with caution because algorithm performances and the agreement between algorithms varies/decreases. Furthermore, the levels of agreement in inertial algorithms were lower in PD cohorts compared to healthy cohorts, suggesting researchers should be more careful while interpreting PD results. Given differences in absolute agreement between algorithms, more efficient and consistent lower-back and shank based IMU algorithms that provide identical results regardless of cohort and environment are needed to use as a powerful tool in clinics, which could be achieved through deep learning approaches. 

## Figures and Tables

**Table 1:** Participant information/experimental protocols.

	DS1			DS2	
*Environment* *Cohort-Number*	*Treadmill* *(YA-16)*	*Indoor* *(YA-31)*	*Outdoor* *(YA-25)*	*Indoor* *(OA-20)*	*Indoor* *(PD-36)*
***Male/Female (n)***	10/6	22/9	16/9	10/10	18/18
***Age(years) Mean ± SD***	32.6 ± 11.9	26.6 ± 11.0	26.28 ± 12.2	69.76 ± 7.82	69.20 ± 6.64
***Sampling Frequency***	60 Hz	60 Hz	75–100 Hz	128 Hz	128 Hz
***Disease Duration (years)***	--	--	--	--	7.82 ± 5.62
***UPDRS III***	--	--	--	--	32.51 ± 4.12
***NFOGQ***	--	--	--	--	7.44 ± 8.62
***LEDD***	--	--	--	--	786.68 ± 416.88

OA: Older Adults, YA: Young Adults, PD: Parkinson’s Disease, UPDRS: Unified Parkinson’s Disease Rating Scale, NFOGQ: The New Freezing of Gait Questionnaire, LEDD: L-dopa equivalent daily dose.

**Table 2:** Extracted temporal parameters and agreements for treadmill walking.

**(YA)** **Treadmill** **DS1** ***n*** **= 16**		**A1-Lower Back**		**A2-Shank**		**Pearson’s R**	**Spearman’s *Rho***	**95% CI Bounds**			
	**Mean Time (s)**	***Average***	***SD***	***Average***	***SD***			***ICC_2,1_***	***Lower***	***Upper***	***p***
	Stride	1.156	0.065	1.152	0.054	0.965 **	0.988 **	**0.975**	0.929	0.991	0.000
	Stance	0.733	0.042	0.732	0.042	0.832 **	0.753 **	**0.914**	0.750	0.970	0.000
	Swing	0.423	0.023	0.420	0.033	0.537 *	0.547 *	**0.684**	0.073	0.890	0.019
	Step	0.578	0.033	0.578	0.027	0.907 **	0.865 **	**0.945**	0.841	0.981	0.000
	**Variability Time (s)**										
	Stride	0.068	0.029	0.075	0.028	0.918 **	0.956 **	**0.946**	0.814	0.982	0.000
	Stance	0.045	0.018	0.084	0.021	0.630 **	0.632 **	0.441	−0,228	0.804	0.005
	Swing	0.026	0.010	0.027	0.006	0.116	−0.300	0.132	−1.666	0.704	0.398
	Step	0.036	0.014	0.040	0.017	0.885 **	0.886 **	**0.915**	0.735	0.971	0.000
	**Asymmetry Time (s)**										
	Stride	0.000	0.000	0.003	0.010	0.436	0.455	**0.564**	−0.150	0.847	0.049
	Stance	0.004	0.004	0.016	0.013	0.019	0.176	0.019	−0.633	0.552	0.476
	Swing	0.004	0.004	0.013	0.008	−0.050	0.037	−0.050	−0.698	0.408	0.563
	Step	0.005	0.005	0.019	0.009	−0.085	0.046	−0.069	−0.509	0.428	0.612

**. Correlation is significant at the 0.01 level (2-tailed). *. Correlation is significant at the 0.05 level (2-tailed).

**Table 3:** Extracted temporal parameters and agreements for indoor walking.

**(YA)** **Indoor** **DS1** ***n*** **= 31**		**A1-Lower Back**		**A2-Shank**		**Pearson’s R**	**Spearman’s *Rho***	**95% CI Bounds**			
	**Mean Time (s)**	***Average***	***SD***	***Average***	***SD***			***ICC_2,1_***	***Lower***	***Upper***	***p***
	Stride	1.096	0.138	1.079	0.138	0.982 **	0.974 **	**0.987**	0.965	0.994	0.000
	Stance	0.692	0.084	0.663	0.092	0.931 **	0.892 **	**0.936**	0.716	0.974	0.000
	Swing	0.402	0.052	0.416	0.058	0.863 **	0.797 **	**0.909**	0.842	0.942	0.000
	Step	0.548	0.069	0.537	0.070	0.989 **	0.984 **	**0.989**	0.916	0.996	0.000
	**Variability Time (s)**										
	Stride	0.040	0.037	0.032	0.018	0.040	0.221 **	0.600	−0.176	0.251	0.294
	Stance	0.026	0.020	0.024	0.015	0.025	0.122 *	0.047	−0.204	0.246	0.343
	Swing	0.019	0.020	0.032	0.011	0.054	0.301 **	0.070	−0.116	0.231	0.217
	Step	0.024	0.021	0.023	0.016	−0.025	−0.016	−0.049	−0.325	0.169	0.656
	**Asymmetry Time (s)**										
	Stride	0.005	0.006	0.007	0.010	−0.034	0.000	−0.060	−0.338	0.159	0.690
	Stance	0.009	0.008	0.016	0.019	0.013	0.800	0.017	−0.214	0.207	0.437
	Swing	0.009	0.009	0.017	0.015	0.130 *	0.155 **	0.184	−0.011	0.344	0.025
	Step	0.011	0.010	0.032	0.036	0.081	0.097	0.062	−0.122	0.223	0.241
** (OA) ** ** Indoor ** ** DS2 ** ***n*** **= 20**	** Mean ** ** Time (s) **										
	Stride	1.162	0.077	1.164	0.0866	0.962 **	0.974 **	** 0.979 **	0.947	0.992	** 0.000 **
	Stance	0.707	0.0404	0.716	0.0630	0.816 **	0.811 **	** 0.851 **	0.631	0.941	** 0.000 **
	Swing	0.447	0.05	0.444	0.0442	0.699 **	0.657 **	** 0.824 **	0.551	0.930	** 0.000 **
	Step	0.579	0.043	0.570	0.0452	0.989 **	0.991 **	** 0.985 **	0.766	0.996	** 0.000 **
	** Variability ** ** Time (s) **										
	Stride	0.086	0.034	0.162	0.106	0.130	0.316	0.124	−0.639	0.603	0.356
	Stance	0.041	0.008	0.151	0.108	−0.153	−0.041	−0.025	−0.494	0.428	0.542
	Swing	0.046	0.012	0.043	0.004	−0.109	−0.039	−0.155	−1.991	0.547	0.621
	Step	0.042	0.010	0.033	0.009	0.061	0.108	0.083	−0.609	0.561	0.396
	** Asymmetry ** ** Time (s) **										
	Stride	0.001	0.002	0.016	0.012	0.147	0.278	0.042	−0.319	0.441	0.418
	Stance	0.000	0.000	0.020	0.016	0.226	0.199	0.013	−0.338	0.406	0.475
	Swing	0.001	0.002	0.012	0.011	−0.028	−0.017	−0.011	−0.549	0.462	0.516
	Step	0.000	0.000	0.016	0.011	0.050	0.068	0.004	−0.177	0.308	0.488
** (PD) ** ** Indoor ** ** DS2 ** ***n*** **= 36**	** Mean ** ** Time (s) **										
	Stride	1.168	0.096	1.183	0.106	0.973 **	0.960 **	**0.979**	0.940	0.991	** 0.000 **
	Stance	0.704	0.051	0.727	0.087	0.804 **	0.750 **	**0.806**	0.608	0.903	** 0.000 **
	Swing	0.458	0.052	0.454	0.052	0.570 **	0.545 **	**0.730**	0.469	0.863	** 0.000 **
	Step	0.584	0.049	0.574	0.049	0.979 **	0.949 **	**0.980**	0.849	0.993	** 0.000 **
	** Variability ** ** Time (s) **										
	Stride	0.083	0.044	0.237	0.161	0.033	0.082	0.018	−0.350	0.360	0.461
	Stance	0.058	0.038	0.231	0.163	0.057	0.315	0.025	−0.295	0.343	0.441
	Swing	0.054	0.023	0.045	0.007	0.316	0.361 *	0.284	−0.299	0.620	0.140
	Step	0.059	0.038	0.038	0.023	0.069	0.525 **	0.097	0.528	0.499	** 0.359 **
	** Asymmetry ** ** Time (s) **										
	Stride	0.002	0.006	0.023	0.021	−0.161	0.136	−0.158	−0.699	0.777	0.760
	Stance	0.001	0.005	0.032	0.024	−0.165	−0.075	−0.062	−0.354	0.256	0.664
	Swing	0.002	0.003	0.026	0.018	−0.309	−0.211	−0.076	−0.343	0.236	0.723
	Step	0.002	0.005	0.033	0.026	−0.200	−0.021	−0.073	−0.391	0.262	0.682

**. Correlation is significant at the 0.01 level (2-tailed). *. Correlation is significant at the 0.05 level (2-tailed).

**Table 4:** Extracted temporal parameters and agreements for outdoor walking.

** (YA) ** ** Outdoor ** ** DS1 ** ***n*** **= 25**		**A1-Lower Back**		**A2-Shank**		**Pearson’s R**	**Spearman’s *Rho***		** 95% CI Bounds **		
	**Mean Time (s)**	***Average***	***SD***	***Average***	***SD***			***ICC_2,1_***	***Lower***	***Upper***	***p***
	Stride	1.084	0.152	1.084	0.153	0.996 **	0.997 **	** 0.998 **	0.997	0.998	** 0.000 **
	Stance	0.680	0.085	0.668	0.111	0.924 **	0.936 **	** 0.940 **	0.913	0.958	** 0.000 **
	Swing	0.403	0.068	0.416	0.055	0.779 **	0.835 **	** 0.856 **	0.790	0.900	** 0.000 **
	Step	0.541	0.076	0.539	0.076	0.996 **	0.993 **	** 0.998 **	0.997	0.999	** 0.000 **
	**Variability Time (s)**										
	Stride	0.025	0.018	0.040	0.030	0.563 **	0.434 **	** 0.605 **	0.314	0.757	** 0.000 **
	Stance	0.018	0.011	0.033	0.026	0.445 **	0.346 **	0.413	0.102	0.607	0.000
	Swing	0.016	0.014	0.035	0.011	0.226 **	0.257 **	0.195	−0.123	0.436	0.004
	Step	0.017	0.011	0.025	0.018	0.044	0.025	0.068	−0.234	0.305	0.314
	**Asymmetry Time (s)**										
	Stride	0.003	0.003	0.006	0.010	0.104	0.202 *	0.109	−0.2013	0.350	0.234
	Stance	0.014	0.014	0.022	0.028	0.079	0.066	0.113	−0.210	0.353	0.226
	Swing	0.014	0.014	0.023	0.024	0.008	−0.026	0.013	−0.337	0.277	0.466
	Step	0.014	0.014	0.040	0.054	0.030	−0.013	0.025	−0.271	0.264	0.429

**. Correlation is significant at the 0.01 level (2-tailed). *. Correlation is significant at the 0.05 level (2-tailed).

## Data Availability

One of the datasets used here can be accessed via http://gaitanalysis.th-brandenburg.de/ (accessed on 1 September 2021). Access to other data can be made to the authors upon reasonable request.

## Supplementary Materials

The following is available online at https://www.mdpi.com/article/10.3390/s21196476/s1, Table S1: Formulas used to calculate temporal parameters along with statistical results.
