# Supplementary material for: Wearable Inertial Gait Algorithms: Impact of Wear Location and Environment in Healthy and Parkinson’s Populations
Source: Sensors (Basel). 2021 Sep 28;21(19):6476. doi: 10.3390/s21196476 (PMC8512498; doi:10.3390/s21196476)
Supplement: Supplementary file 1 [file sensors-21-06476-s001.zip › sensors-1384979-supplementary.pdf]

sensors

MDPI

Supplementary Materials

Algorithm 1 (Pseudocode)

1: Filter raw vertical hip acceleration signal (The 4th order Butterworth filter with 10 Hz cut off frequency)
2: Acceleration correction to horizontal-vertical frame [33]
3: Integrated_av: Numerical integration of vertical acceleration of hip in the horizontal-vertical frame (cumtrapz)
4: IC_moments = Gaussian continuous wavelet transforms at scale 10 (Integrated_av)
5: FC_moments= Further differentiation of IC_moments
6: **for** i=1: size (IC_moments)
7: IC=find local minima in IC_moments
8: **end for**
9: **for** i=1: size (FC_moments)
10: FC=find local maxima in FC_moments
11: **end for**
12: **Return** IC and FC

Algorithm 2 (Pseudocode)

1: Wavelet decomposition (5th order coiflet, at scale 10) of shin sagittal plane angular velocity
2: Get two new approximations (a1 and a2) with appcoef function
3: ms_a1= Find peaks in a1 to locate mid swing
4: ms_a2= Find peaks in a2 to locate mid swing
4: **for** i=1: size (a1)
5: IC=find local minima in the range of [ms_a1+0.25s, ms_a1+2s]
6: **end for**
7: **for** i=1: size (a2)
8: FC=find local minima in the range of [ms_a2-2s, ms_a2-0.05s]
9: **end for**
10: **Return** IC and FC

Note: step time statistical formula is presented in Table S1 only, the same formulas are used for the remaining temporal outcomes.

Table S1. Formulas used to calculate temporal parameters along with statistical results

	**Temporal parameter formula (left side only)**
**Algorithm 1 (A1)**	Stride Time= IC(i+2) - IC(i), Stance Time= FC(i+1) - IC(i), Swing Time= IC(i+2) - FC(i+1), Step Time= IC(i+1)- IC(i)
**Algorithm 2 (A2)**	Stride Time= IC(j+2) - IC(j), Stance Time= FC(j+1) - IC(j), Swing Time= IC(j+2) - FC(j+1), Step Time= IC(j+1)- IC(j)
	**Statistical formulas (both sides-for Step Time only)**
**Mean**	$\left(\text{mean(Step time}_{\text{left}}) + (\text{Step time}_{\text{right}})\right)/2$
**Variability**	$\sqrt{\left(\text{var}\left(\text{Step time}_{\text{left}}\right) + \text{var}\left(\text{Step time}_{\text{right}}\right)\right)/2}$
**Asymmetry**	$\left\|\text{mean}\left(\text{Step time}_{\text{left}}\right) - \text{mean}\left(\text{Step time}_{\text{right}}\right)\right\|$
